# Haematological and biochemical abnormalities in hunting dogs infected with *Acanthocheilonema reconditum*, associated risk factors, and a European overview

**DOI:** 10.1007/s00436-021-07179-8

**Published:** 2021-05-08

**Authors:** Laura Pacifico, Nicola Ferrari, Claudia Romeo, Francesco Buono, Paolo Varuzza, Giovanni Sgroi, Benedetto Neola, Jesse Buch, Melissa Beall, Edward B. Breitschwerdt, Ramaswamy Chandrashekar, Vincenzo Veneziano, Diego Piantedosi

**Affiliations:** 1 Department of Veterinary Medicine and Animal Productions, Università degli Studi di Napoli “Federico II”, Via F. Delpino 1, 80137 Naples, Italy; 2 Department of Veterinary Medicine, Università degli Studi di Milano, Via Celoria 10, 20133 Milan, Italy; 3 Department of Veterinary Medicine, University of Bari Aldo Moro, Valenzano, Bari, Italy; 4 IDEXX Laboratories, Inc., Westbrook, ME 04092 USA; 5 Department of Clinical Sciences, College of Veterinary Medicine, North Carolina State University, Raleigh, NC 27607 USA; 6 Osservatorio Faunistico Venatorio - Regione Campania, Naples, Italy

**Keywords:** Filarial nematodes, *Acanthocheilonema* spp., CVBDs, Hunting dogs

## Abstract

*Acanthocheilonema reconditum* is a filarial parasite transmitted by arthropods (fleas, lice, and ticks) that infect dogs. There is minimal published data available to date on potential haematological and biochemical changes associated with this parasitic infection. Study aims were (i) provide an overview of *A. reconditum* in Europe, (ii) define *A. reconditum* prevalence and risk factors in a specific dog population (hunting) from southern Italy, and (iii) assess the frequency of haemato-biochemical abnormalities associated with infection. Blood samples collected from 3020 dogs were tested by a modified Knott’s technique to count and identify microfilariae. Eighty-four dogs were infected by *A. reconditum* (2.78%; 95% CI 2.19–3.37%). Microfilariae ranged from 1 to 212/ml. Based on clinical examination, all but six dogs with non-specific symptoms were healthy. Haematological abnormalities included leucocytosis (*n* = 15), with eosinophilia (*n* = 14) and monocytosis (*n* = 13). Serum biochemical abnormalities included increased total serum proteins (*n* = 19), albumins (*n* = 7), total globulins (*n* = 14), ALT (*n* = 1), and ALP (*n* = 1); one dog was hypoalbuminemic, and BUN was mildly increased in 2 dogs. Risk factors included the province origin (Napoli, OR=5.4, 95%CI: 2.1–14.0; Caserta, OR=5.1, 95%CI: 2.5–10.6), hunting wild mammals (OR=2.8, 95% 95%CI: 1.6–4.8), and ectoparasite infestation (OR=1.9, 95%CI: 1.1–3.1). There was a negative correlation between microfilaraemic load and decreased albumin level (−0.37; *p*=0.021). Our results showed that *A. reconditum* circulates within the hunting dog population of southern Italy, with seemingly low pathogenic potential.

## Introduction

Canine vector-borne diseases (CVBDs) are of historical and growing concern in veterinary and human medicine, and among these, particular interest is focused on the spread, diagnosis, and control of filarial worms (Otranto et al. 2013; Capelli et al. 2018; Genchi and Kramer 2020). Filarial nematodes reported in dogs include *Dirofilaria immitis*, *Dirofilaria repens*, *Acanthocheilonema reconditum* (syn. *Dipetalonema reconditum*), *Acanthocheilonema dracunculoides*, whose microfilariae parasitize the blood, and *Cercopithifilaria grassi*, *Cercopithifilaria bainae*, *Cercopithifilaria* sp. II *sensu* Otranto et al. 2012, and *Onchocerca lupi*, whose larval forms localize within the subcutaneous tissue (Ramos et al. 2016)*.* Due to their pathogenic potential, *D. immitis* and *D. repens* represent the most well-studied species, while knowledge about other filarial nematodes such as *A. reconditum* is considerably less well characterized (Dantas-Torres and Otranto 2020; Genchi and Kramer 2020; Panarese et al. 2020)*.*

*Acanthocheilonema reconditum* (syn. *Dipetalonema reconditum*) parasitizes domestic and wild canids, such as foxes (Marconcini et al. 1996; Ionică et al. 2017; Otranto et al. 2019). Reported intermediate hosts include fleas (*Ctenocephalides canis*, *Ctenocephalides felis*, *Pulex irritans*, *Pulex simulans*, *Echidnophaga gallinae*), lice (*Heterodoxus spiniger*, *Linognathus setosus*), and ticks (*Rhipicephalus sanguineus*) (Cringoli et al. 2001; Brianti et al. 2012; Napoli et al. 2014). *Acanthocheilonema reconditum* is widespread and its presence has been reported in Asia, Africa, America, Oceania, and Europe, localized mainly in the Mediterranean area (Otranto et al. 2013); furthermore, it is reported historically as the most common filaroid species in southern Italy, as well as *D. repens* (Mendoza-Roldan et al. 2020).

*Acanthocheilonema reconditum* is commonly considered an apathogenic parasite, as most studies do not describe clinical disease associated with confirmed infection (Pantchev et al. 2011; Brianti et al. 2012; Otranto et al. 2013). In addition, Papazahariadou et al. (1994) reported no correlation between episodic weakness in hunting dogs and *A. reconditum* infection. In contrast, a purulent mesenteric lymphadenitis was reported by Lindemann et al. (1983) as acute response in an experimentally infected dog. Martins Pereira et al. (2004) also described two erratic migrations and ectopic localizations of adult worms, with one dog having adult parasites in the heart (left ventricle), in association with bronchopneumonia and microfilariae in the alveoli, and the other with adult worms in the ovarian large ligament, occasionally found during ovariectomy. Other sporadic clinical abnormalities related with *A. reconditum* infection included subcutaneous suppurative inflammatory nodules in a naturally infected dog (Engelmann et al. 2019). Regarding clinical pathology, few reports have considered the occurrence of haematological and biochemical abnormalities in *A. reconditum-*infected dogs. A peripheral eosinophilia was found in experimentally infected dogs in the acute/invasive phase, while lymphocytosis developed during the chronic phase of infection (Lindemann et al. 1983). In one study involving naturally infected dogs, Hashem and Badawy (2007) reported regenerative hypochromic anaemia, leucocytosis with neutrophilia, eosinophilia and monocytosis, increased serum liver enzymes (aspartate aminotransferase-AST, alanine aminotransferase-ALT and total bilirubin), and renal function parameters (blood urea nitrogen-BUN, creatinine, and serum inorganic phosphate).

Hunting dogs are frequently exposed to vector-borne pathogens due to their lifestyle, which is characterized by closer contact with wooded areas, cohabitation in outdoor kennels, and potentially less consistent use of antiparasitic drugs with limited care provided by owners (Piantedosi et al. 2017; Orr et al. 2020). Moreover, intense physical activity makes hunting dogs more susceptible to episodic weakness, as often reported by hunters. Diagnostic evaluation of episodic weakness can be challenging, and a previous study associated the presence of filariasis with exercise intolerance (Papazahariadou et al. 1994). While the pathogenesis of *D. immitis* microfilariae is well known, minimal data regarding the pathogenic potential role of *A. reconditum* microfilariae are available (McCall et al. 2008; Muñoz-Caro et al. 2018).

Based on the above premises, the aims of the present study were (i) to illustrate the distribution and the epidemiology of *A. reconditum* infection in dogs in Europe, (ii) to assess the prevalence and risk factors of *A. reconditum* infection in a large and specific hunting dog population from southern Italy, and (iii) to determine the frequency of haematological and serum biochemical abnormalities associated to *A. reconditum* infection as related to the microfilariae load.

## Overview of *Acanthocheilonema reconditum* epidemiology in dogs in Europe

*Acanthocheilonema reconditum* (syn. *Dipetalonema*) was first described by Grassi in 1889. Grassi and Calandruccio (1890) defined morphological differences with *D. immitis*, by reporting details on an immature female worm; the same authors proved that *A. reconditum* develops in fleas. Additional data was reported by Noë (1907), who described further morphological features, analyzing some adult specimens (male and female) of *A. reconditum*. Additional morphological characteristic of *A. reconditum* microfilariae was subsequently reported by Newton and Wright (1956). Subsequent studies further defined the life cycle and completed the description of the parasite’s morphology (Newton and Wright 1956; Nelson 1962; Bain and Beaucournu 1974; Korkejian and Edeson 1978). *Acanthocheilonema reconditum* infections are reported in several European countries, and this worm is considered the main filaroid species infecting dogs in the Mediterranean basin (Brianti et al. 2012). Otranto et al. (2013) reported the geographical distribution of *A. reconditum*, showing its presence in Italy, Spain, Germany, Austria, and Greece. In another recent review, Tahir et al. (2019) described *Acanthocheilonema* infections (*A. reconditum* and *A. dracunculoides*), reporting the distribution in dogs and foxes in the Mediterranean basin, including the Middle East and northern Africa.

Full information about the prevalence of this parasite in dogs in Europe is scarce; most often its occurrence is a secondary finding within epidemiological surveys focused on other major Filaridae, such as *D. immitis* or *D. repens*.

In Italy, the presence of *A. reconditum* has been confirmed by different surveys in southern areas of the country (mean prevalence 7.7 %) (Cringoli et al. 2001; Veneziano 2001; Giannetto et al. 2007; Brianti et al. 2012). In northern and central regions of Italy, a lower prevalence was reported (mean prevalence 1 %) (Veneziano 2001; Magi et al. 2012; Gizzarelli et al. 2019; Macchioni et al. 2020), but in contrast, a higher infection rate (7.7 %) was described in Liguria region by Magi et al. (2016). Two recent countrywide Italian surveys reported an overall *A. reconditum* prevalence of 0.8% and 0.1% (Brianti 2018¸ Traversa et al. 2019).

Among the other Mediterranean basin countries, presence of *A. reconditum* has been described in Greece, Cyprus, Turkey, Spain, Portugal, and France. In Greece, most studies are from the northern area of the country with a mean prevalence of 2.4 % (Papazahariadou et al. 1994; Founta et al. 1999; Diakou et al. 2016). In Cyprus, *A. reconditum* was the most represented among microfilaria species (Attipa et al. 2019; Kokkinos et al. 2019). In Turkey, Toparlak et al. (2005) showed *A. reconditum* as the only microfilaria species with a low prevalence (0.6 %) compared to the other Mediterranean countries. In Spain, a mean prevalence of 1.3 % was reported (Perez-Sanchez et al. 1989; Ortega-Mora et al. 1991; Aranda et al. 1998), with hotspots in a central-northern area of the country (Soria province—15.8%) and in the Canary Islands (20%) (Ortega-Mora et al. 1991) In the Iberian Peninsula, Portugal showed a lower prevalence, with a mean value of 0.55 % (Menn et al. 2010; Maia et al. 2016; Ferreira et al. 2017).

In France, the presence of *A. reconditum* was reported in different vector and life cycle studies, which contributed to the definitive classification of filarial species (Bain and Beaucournu 1974; Chauve 1990; Chabaud and Bain 1994). A very low prevalence (0.05%) was reported by Ducos de Lahitte in 1990. Despite several studies about filariosis, to the best of our knowledge, there are no recently published data on the prevalence of *A. reconditum* in France, except for a 2019 survey reporting negative results for *A. reconditum* infection (Laidoudi et al. 2019).

Regarding countries of central Europe, in Switzerland, Belgium, and the Netherlands, despite the ascertained presence of other filarial species, *A. reconditum* seems never to have been reported (Meyer et al. 1994; Deplazes et al. 1995; Bucklar et al. 1998; Petruschke et al. 2001; Overgaauw and van Dijk 2009; De Bosschere and Kindermans 2019).

Several studies involving *A. reconditum* have been carried out in Germany with most of the infections described in imported dogs (Zahler et al. 1997; Pantchev et al. 2011; Hamel et al. 2012; Schäfer et al. 2019), but only few autochthonous cases were reported in central Germany (0.19 %) (Liesner et al. 2016).

In Austria, a single *A. reconditum* imported case was documented (Hinaidy et al. 1987), but no autochthonous infections were described (Prosl et al. 2003; Duscher et al. 2009).

In the UK, there is only one dated study describing *A. reconditum* microfilariae in imported dogs from Ireland (Jacobs and Prole 1976).

In the Balkanian area, the presence of *A. reconditum* was described in the northern region of Serbia (2.1 %) (Tasić et al. 2008), although two subsequent studies in the same country found negative results (Tasić et al. 2012; Potkonjak et al. 2020). In Slovenia, Croatia, and Bulgaria, *A. reconditum* microfilaraemia was not detected in surveys on filariosis in dogs (Kirkova et al. 2007; Georgieva et al. 2001; Morchón et al. 2012; Holler et al. 2010; Radev et al. 2016).

In the eastern European countries, *A. reconditum* was reported in Romania (2.05 %) (Ionică et al. 2015), while in Slovakia, the parasite was not found (Miterpáková et al. 2010; Víchová et al. 2014). In Poland, *A. reconditum* was reported in 2011, when two dogs were found positive during a survey on other filarial species (Masny et al. 2011).

**Fig. 1:**
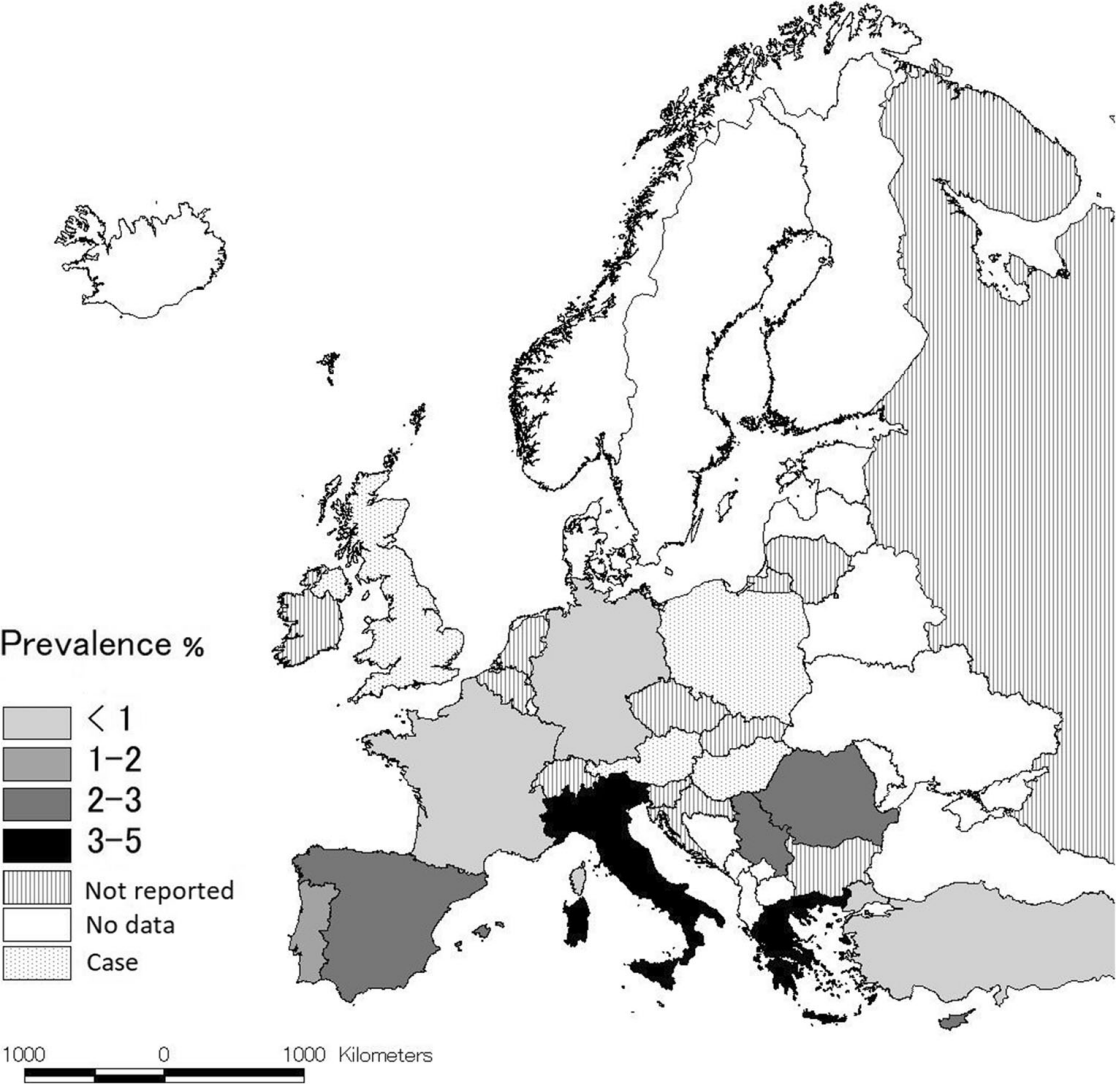
Pooled prevalence (%) of *Acanthocheilonema reconditum* in dogs within the European Union and adjacent countries at national level

In Baltic countries, despite the high prevalence of *D. repens* (Alsarraf et al. 2021), *A. reconditum* infection was not reported (Kartashev et al. 2011; Sabūnas et al. 2019) (Table 1; Fig. 1).

**Table 1:** Epidemiological surveys and reports on prevalence of *Acanthocheilonema reconditum* in dogs in Europe

Country*	Region/area	No° of tested dogs	Dog population	N° of *A. reconditum* positive/prevalence	Methods	References
Mediterranean area						
Italy	Northern regions	11,782	General dog population	169 (1.4%)	Knott test	Veneziano 2001 (PhD Thesis)
	Central regions	1624	General dog population	3 (0.2%)	Knott test	Veneziano 2001 (PhD Thesis)
	Southern regions	1157	General dog population	58 (2.3%)	Knott test	Veneziano 2001 (PhD Thesis)
	Sicily-Sardinia	428	General dog population	101 (2.3%)	Knott test	Veneziano 2001 (PhD Thesis)
	Campania	351	General dog population	58 (16.5%)	Knott test	Cringoli et al. 2001
	Sicily	2,512	Owned	114 (4.5%)	Knott test	Giannetto et al. 2007
	Sicily	152 (in 2010) 120 (in 2011)	Stray-kennel	17 (11.2%) 16 (13.3%)	Knott test Knott test	Brianti et al. 2012
	Tuscany	630	Kennel	12 (1.9%)	Knott test-acid phosphatase activity	Magi et al. 2012
	Liguria	365	Hunting-kennel-owned	40 (7.7 %)	Knott test-acid phosphatase activity-PCR	Magi et al. 2016
	Countrywide	1748	General dog population	0.8%	Knott test	Brianti 2018
	Molise	990	Hunting-stray-sheepdog	10 (1.3%)	Knott test	Gizzarelli et al. 2019
	9 different regions (north, central and southern)	1055	General dog population	1 (0.1%)	Knott test	Traversa et al. 2019
	Tuscany-Lazio	363	Kennel-hunting	3 (0.1%)	Knott test	Macchioni et al. 2020
Greece	Thessaloniki	100	Hunting	6 (6%)	Knott test	Papazahariadou et al. 1994
	Thessaloniki	252	General dog population	10 (3.9%)	Knott test	Founta et al. 1999
	Thessaloniki-Larissa-Attica-Achaia-Herklion	750	Owned	10 (1.3%)	Knott test	Diakou et al. 2016
Cyprus	Pafos-Lamessos-Larnaka-lefkosia-Ammochostos	200	Owned - Kennel	9 (4.5%)	Knott test - PCR	Kokkinos et al. 2019
	Pafos	134	Owned	2 (1.49%)	PCR	Attipa et al. 2019
Turkey	Istanbul	286	Stray	2 (0.6%)	Acid phosphatase activity	Toparlak et al. 2005
	Kayseri province	280	Stray-owned	Not reported	Acid phosphatase activity	Yildirim et al. 2007
	Erzurum, northeastern Turkey	133	Kennel	Not reported	PCR	Guven et al. 2017
Spain	Salamanca province	293	Work–sport	6 (2.1%)	Knott test-acid phosphatase activity	Perez-Sanchez et al. 1989
	Countrywide	1723	Owned	17 (1.0%)	Knott test	Ortega-Mora et al. 1991
	Baix Llobregat area, Barcellona	188	Owned-rural	7 (3.7%)	Knott test-acid phosphatase activity	Aranda et al. 1998
Portugal	Not specified	331	Owned	6 (1.81%)	Knott test-acid phosphatase activity	Menn et al. 2010
	Southern regions	230	Owned-kennel	1 (0.4%)	PCR	Maia et al. 2016
	Coimbra, Santarém and Setúbal areas	878	Kennel	2 (1.5%)	Knott test-acid phosphatase activity	Ferreira et al. 2017
	Coimbra, Santarém and Setúbal areas	720	Kennel	0.4	PCR	Ferreira et al. 2017
France	Countrywide	5503	General dog population	3 (0.05%)	Knott test	Duchos de Lahitte 1990
	Indre department	17	Kennel	Not reported	PCR	Laidoudi et al. 2019
Central and Balkan countries						
Switzerland	Southern areas	479	Owned	Not reported	Acid phosphatase activity	Bucklar et al. 1998
	Canton Ticino	371	Stray-kennel	Not reported	Difil test	Deplazes et al. 1995
	Southern areas	308	General dog population	Not reported	-	Petruschke et al. 2001
Austria	Case report	1	-	Reported	Knott test	Hinaidy et al. 1987
	Imported from Mediterranean area	87	-	Not reported	Knott test	Prosl et al. 2003
	Imported dogs	45	General dog population	Not reported	Knott test	Leschnik et al. 2008
	Gänserndorf Neusiedl, southern district	96	General dog population	Not reported	Knott test-PCR	Duscher et al. 2009
Hungary	Countrywide except Budapest	344	General dog population	Not reported	Knott test-PCR	Farkas et al. 2020
Germany	Imported dogs	80	-	3 from Spain and Corsica	Knott test-acid phosphatase activity	Zahler et al. 1997
	Imported dogs	8545	Owned	12 from Spain 1 from Hungary	Knott test-PCR	Pantchev et al. 2011
	Imported dogs	216	Owned-stray	1 from Romania	PCR	Hamel et al. 2012
	Brandeburg federal state	1023	Owned	2 (0.19%)	PCR	Liesner et al. 2016
	Imported dogs	178	-	1 from Spain	Knott test	Schäfer et al. 2019
Netherlands	Imported dogs	7	-	Not reported	Knott test	Meyer et al. 1994
	Case report	1	Owned	Not reported	Knott test	Overgaauw and van Dijk 2009
Belgium	Case report	1	Owned	Not reported	-	De Bosschere and Kindermans 2019
UK	Imported from Ireland	270	Racing	15 (5.6%)	Acid phosphatase activity	Jacobs and Prole 1976
Serbia	Vojvodina	193	Owned	4 (2.1%)	Knott test	Tasić et al. 2008
	Northern area	122	General dog population	Not reported	Knott test-PCR	Tasić et al. 2012
	Vojvodina	59	Stray	Not reported	PCR	Potkonjak et al. 2020
Slovenia	-	-	-	Not reported	-	Morchón et al. 2012
Croatia	Buzeština	200	Hunting-owned	Not reported	Knott test	Holler et al. 2010
Bulgaria	Not specified	258	Working-shepherd-rural-stray-owned	Not reported	Knott test	Georgieva et al. 2001
	Countrywide	487	Owned	Not reported	Knott test	Kirkova et al. 2007
	Sofia and Pleven district	33	Stray-kennel	Not reported	Knott test	Radev et al. 2016
Poland	Case report	28	Owned	2	Knott test - PCR	Masny et al. 2011
Eastern and northern countries						
Romania	Southern-east regions	390	Owned	8 (2.05%)	PCR	Ionică et al. 2015
Slovakia	Countrywide	710	Working dogs	Not reported	Knott test	Miterpáková et al. 2010
	Countrywide	366	Working- Owned- Hunting - Stray	Not reported	Knott test - PCR	Víchová et al. 2014
Czech Republic	Not specified	93	Imported and local dogs	Not reported	Knott test	Svobodova and Misonova 2005
	Southern regions	77	General dog population-hunting	Not reported	Knott test-acid phosphatase activity-PCR	Svobodová et al. 2006
Lithuania	Central - East regions	2280	Owned-kennel	Not reported	Knott test-PCR	Sabūnas et al. 2019
Russia	Rostov region	795	General dog population	Not reported	Knott test	Kartashev et al. 2011

^*^Countries have been regrouped according to climatic areas as suggested by Capelli et al. (2018).

## Materials and methods

### Study area

The study area included six different hunting districts (ATCs) in Campania and Basilicata regions of southern Italy. These are located in the provinces of Napoli (40° 50′ N-14° 15′ E) (ATC NA), Avellino (40° 54′ 55″ N-14° 47′ 22″ E) (ATC AV), Benevento (41° 08′ N-14° 47′ E) (ATC BN), Caserta (41° 10′ N-14° 13′ E) (ATC CE), Salerno (40° 41′ 00″ N-14° 47′ 00″ E) (ATC SA1), and Potenza (40° 38′ 19″ N-15°48′ 08″ E) (ATC PZ). The territory of the Napoli, Caserta, Salerno, and Potenza provinces overlooks the Tyrrhenian Sea. The study region has a typical Mediterranean temperate climate along the coast, which becomes progressively continental in the inland and mountainous territories.

### Study animals and sample size

The survey included 3020 hunting dogs and was performed in 81 private veterinary clinics located in the study area from 2014 to 2019; all procedures were performed for diagnostic purposes during a routine health check, and for this reason, the study did not require an ethical approval according to European Directive 2010/63/EU. Written informed consent was obtained from the owners of the hunting dogs included in the study.

The study was performed within a hunting dog’s health assistance program of University of Naples, supported by the Italian management committees of the respective hunting districts (ATCs). In order to rule out the spread of major canine vector-borne pathogens in the study area, serological in-clinic assay test systems [SNAP®4Dx®Plus (*Ehrlichia canis*, *Anaplasma* spp., *Borrelia burgdorferi* s.l., *D. immitis*)], based on enzyme immunoassay technique, were employed on each dog enrolled. Additionally, *A. reconditum* microfilaremic dogs were tested for antibodies to *Leishmania infantum* and for the presence of *Angiostrongylus vasorum* antigen, using the SNAP® Leishmania and IDEXX Angio Detect® Test kits, respectively, in order to rule out a possible coinfection with these two pathogens.

To better define *A. reconditum* infection risk, a questionnaire was administered to each owner to collect information about the dog’s characteristics, habits, and management/handling that might influence exposure and/or susceptibility to the parasite (see “Statistical analysis” for details). Because hunters from the Campania region have the habit of extra-label use of subcutaneous ivermectin injection for the prevention of ecto- and endoparasite infections (Piantedosi et al. 2017), owners were asked specifically in the questionnaire about macrocyclic lactone treatment schedule.

The necessary sample size to estimate prevalence of *A. reconditum* was calculated using the formula proposed by Thrusfield (1995), considering the following epidemiological data: expected prevalence of 16% based on the results of a similar study in no specific dog population from Campania region (southern Italy) (Cringoli et al. 2001); confidence interval (99%) and desired absolute precision (2%), based on the number of hunters in Campania region (*n*° 38,611 hunters in the season 2014–2015 and assuming one dog for each hunter) (BURC 2019).

### Sample collection

For each dog, blood samples were obtained through percutaneous venipuncture of cephalic or jugular veins, after 12h of fasting. The total amount of blood was immediately divided into three aliquots. The first two aliquots were placed in tubes containing potassium ethylene diamine tetra-acetic acid (EDTA) for complete blood count (CBC) and Knott test, which were carried out within 30 min of blood collection. The third aliquot was put in tubes without anticoagulant, allowed to clot and centrifuged at 908*g* for 15min at 4°C, in order to obtain blood serum samples that were stored at −80°C and defrosted immediately before batch analyses of haematochemical parameters.

### Parasitological analysis

For each dog involved in the study, the Knott’s test technique (Knott, 1939) was carried out at the parasitology laboratories of the Department of Veterinary Medicine and Animal Productions, University Federico II, Naples, to find microfilariae in blood as follows: 1 ml of whole blood was diluted with 9 ml of 2% formalin in a centrifuge tube and after centrifugation for 10 min at 1500 rpm the supernatant was discarded. Sediment sample (20 μl) was transferred to a glass slide up to complete 1 ml and examined using optical microscope at 10, 20, 40, and ×100 magnifications (Genchi et al. 2010).

In order to differentiate *A. reconditum* from other filarial species, blood microfilariae (*D. repens*, *D. immitis*, *A. dracunculoides*) were identified by morphological and morphometrical examinations. The diagnostic keys used to differentiate microfilariae species were morphology, shape of larval head and tail, and size measurements of length and width according to McCall et al. (2008). In addition, microfilariae were counted, and numbers were expressed as microfilariae/ml of blood (mf/ml blood). All the diagnostic procedures were performed with a LEICA DM 750 microscope (Germany) with digital camera; the image analysis system Leica Application Suite V 4.9 (LAS – ®Leica Microsystem) was used to get microfilariae measurements and take pictures.

### Clinical examination, complete blood count, and serum biochemistry

A clinical examination was performed on each *A. reconditum* microfilaremic dog to evaluate the presence of systemic clinical signs such as fever, lymphadenomegaly, splenomegaly, changes in mucous membrane colour, cardiorespiratory abnormalities, skin lesions, and dehydration. The nutritional status was assessed using a nine-point body condition score (BCS) system (Laflamme 1997). A complete blood count (CBC) and a basic biochemical panel were obtained on each positive animal at the clinical pathology laboratories of the Department of Veterinary Medicine and Animal Productions, University Federico II, Naples.

Complete blood counts were performed on 32 microfilaremic dogs using a semi-automatic cell counter (HM5, Abaxis, USA). An automatic chemical chemistry analyzer (VetScan Vs2, Abaxis, USA) was used to measure concentrations or activities of blood urea nitrogen (BUN), creatinine, alanine aminotransferase (ALT), alkaline phosphate (ALP), total serum proteins (TP), albumins, and total globulins (TG) for 43 microfilaremic dogs.

### Statistical analysis

Factors influencing the probability for dogs of being infected by *A. reconditum* were explored through a mixed effects logistic regression model with binary outcome. Ninety subjects were removed from the analysis due to missing information about one or more of the considered explanatory variables, for a final sample size of 2930 dogs. The effect on *A. reconditum* infection status (infected or uninfected) of the following variables extracted from the owners’ questionnaire was examined: dog’s age, gender, type of coat (short, medium, or long hair), living area (province) and environment (rural or urban), night shelter availability (yes or no), pack size, number of hunting months, type of hunted species (birds or wild mammals), travel abroad (yes or no), reported ectoparasite infestation history (yes or no), and ectoparasite treatment (yes or no). Owners’ IDs were included in the model as a random factor, to account for potential covariance within packs. We first fitted a full, saturated model including all factors and obtained a minimal model through backward elimination of non-significant variables. Odds ratio (OR) estimates of the significant factors retained in the minimal model and their 95% confidence intervals (CIs) are presented.

For dogs with only *A. reconditum* infection, a Pearson’s correlations was used to examine the association of the measured microfilarial load with the following haematological and biochemical variables: red blood cell count (RBC), haemoglobin concentration (Hb), mean corpuscular haemoglobin (MCH), mean corpuscular haemoglobin concentration (MCHC), white blood cell count (WBC) and percentage, absolute value of lymphocytes, neutrophils, eosinophils, and monocytes, and serum albumin level. All the analyses were carried out using SAS/STAT 9.4 software (Copyright © 2012, SAS Institute Inc., Cary, NC, USA).

## Results

**Fig. 2:**
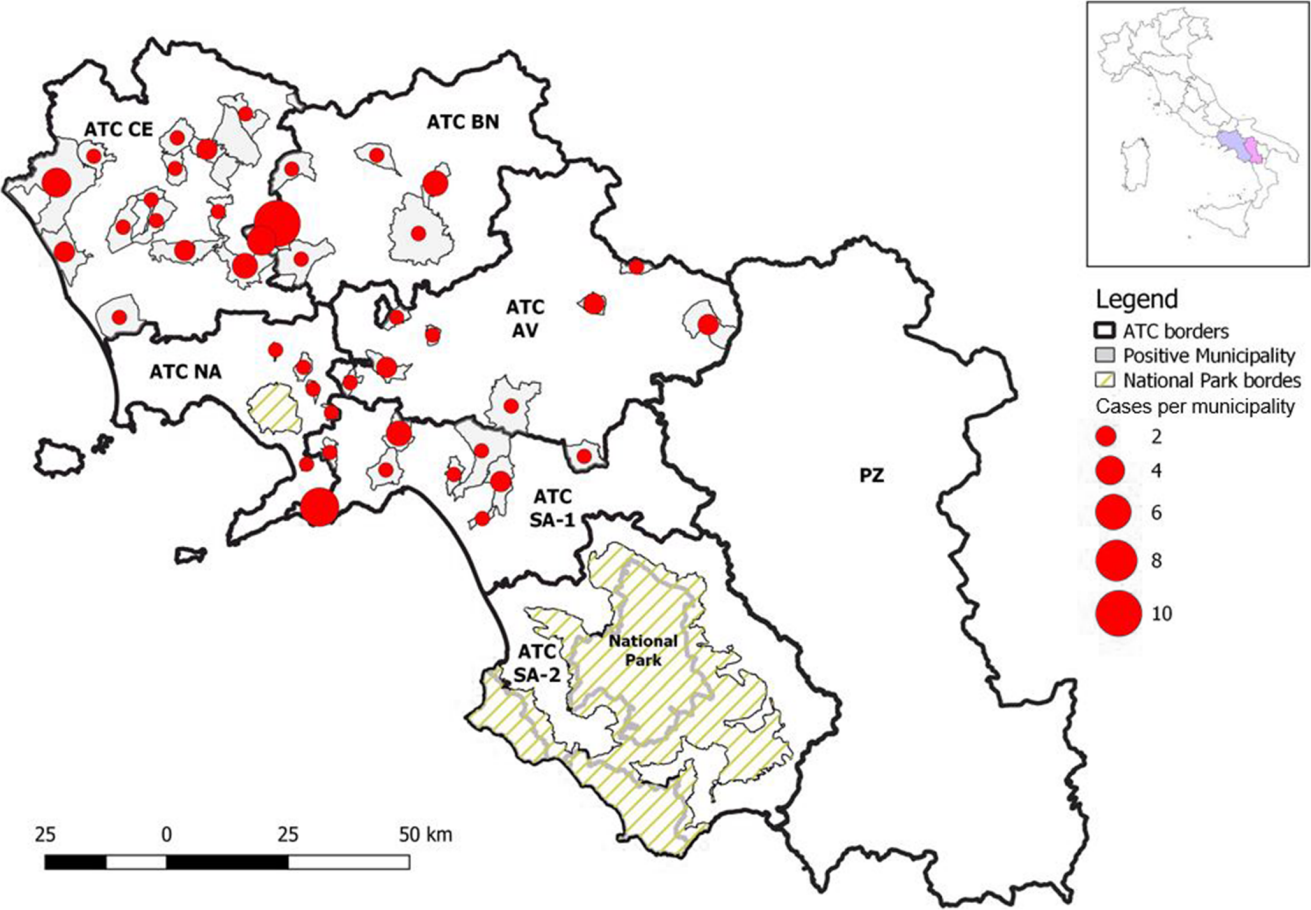
Distribution map of *Acanthocheilonema reconditum* positive hunting dogs in the study area

Of the 3020 dogs, 1660 were males (55%) and 1360 (45 %) females; average age was 3.8 years (min 0.25-max 14). A total of 84 dogs were positive to *A. reconditum* at the Knott’s test, with an overall prevalence of 2.8% (95% CI: 2.19–3.37) (Fig. 2). A monospecific *A. reconditum* infection was detected in 71 dogs, whereas 4 dogs had mixed filarial infections (3 co-infected with *D. repens* and 1 with *D. immitis*); the remaining 9 dogs were co-infected with other vector-borne pathogens, prevalent in southern Italy (3 co-infected with *Leishmania infantum*; 3 co-infected with *Ehrlichia canis* and 3 co-infected with *E. canis* and *Anaplasma* spp., respectively).

Of the 71 dogs with only *A. reconditum* infection, 35 (48.6%) were from Caserta, 12 (16.9%) from Napoli, 12 (16.9%) from Avellino, 6 (8.5%) from Benevento, and 6 (8.5%) from Salerno province. No dogs from Potenza province had *A. reconditum* infection.

**Fig. 3:**
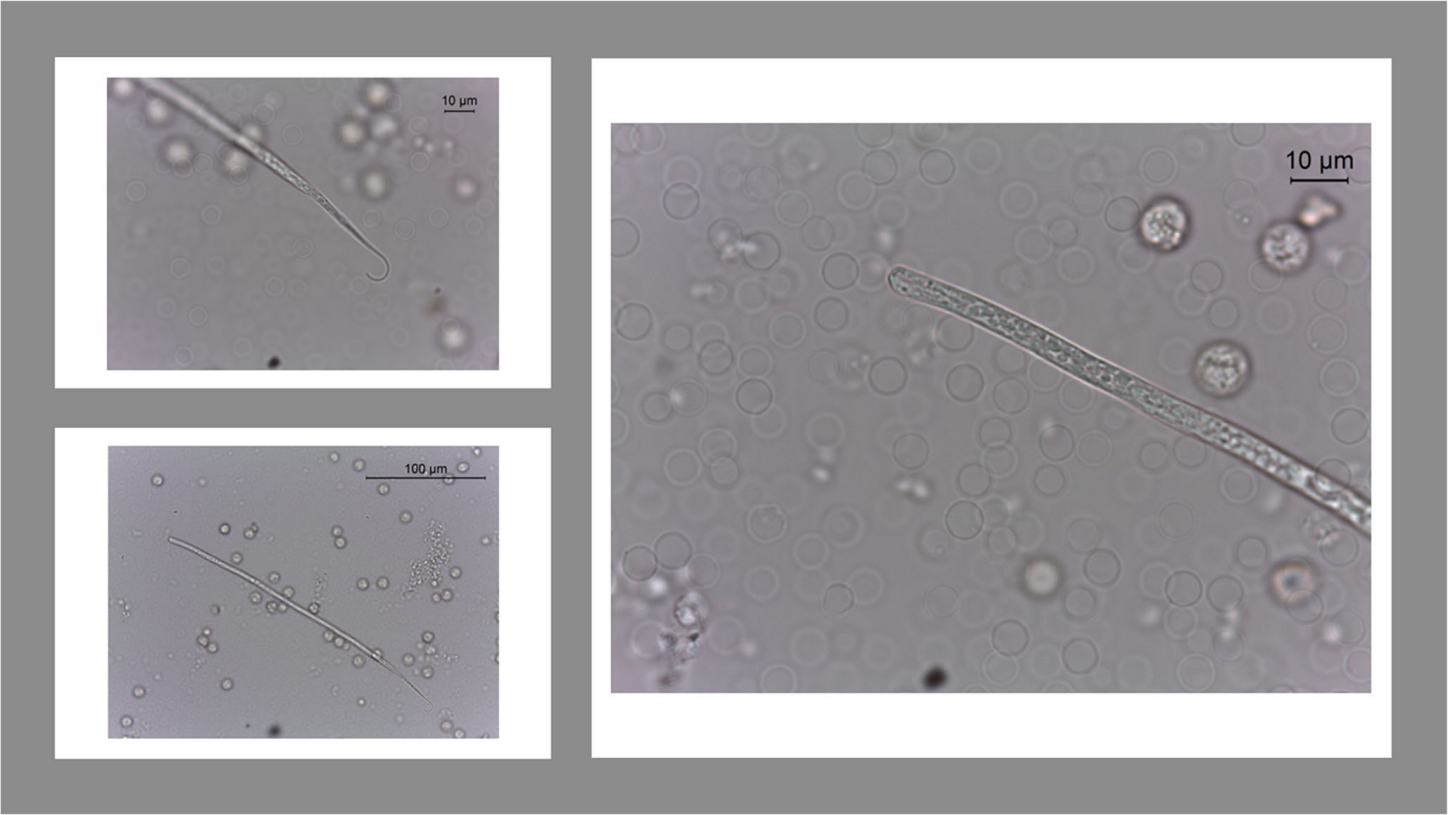
Morphological details of *Acanthocheilonema reconditum microfilariae isolated by the Knott test *

Based upon microscopic microfilariae counts, dogs were infected with an average ± standard deviation (SD) of 29 ± 36 microfilariae/ml (min 1-max 212); the mean length was 265.7 μm ± 14.1 and the mean width was 4.9 μm ± 0.5 (Fig. 3).

During clinical examinations, the following clinical signs and laboratory abnormalities were reported in 71 *A. reconditum* single infection microfilaremic dogs: dehydration (*n* = 1), fever (*n* = 1), congested mucous membranes (*n* = 1), weight loss (BCS 3) (*n* = 1), and exercise intolerance (*n* = 2). CBC results revealed normocytic hypochromic anaemia (*n* = 1), thrombocytopenia (*n* = 7), thrombocytosis (*n* = 2), leukocytosis (*n* = 15), lymphocytosis (*n* = 4), lymphopenia (*n* = 6), neutropenia (*n* = 11), basophilia (*n* = 1), monocytosis (*n* = 13), and eosinophilia (*n* = 14). Of the 7 thromobocytopenic dogs, 4 had platelet aggregation on microscopic blood smear examination. The serum biochemical abnormalities (serum values above the upper limit of the reference range) included increased total serum proteins (*n* = 19), albumins (*n* = 7), total globulins (*n* = 14), ALT (*n* = 1), and ALP (*n* = 1); one dog was hypoalbuminemic. Six dogs had BUN values below the reference range, and 2 dogs slightly above the reference interval. The mean value (±SD) of haematological and biochemical data are resumed in Table 2 and Table 3.

**Table 2:** Mean value for complete blood count results in *Acanthocheilonema reconditum* microfilaremic dogs

Parameter	Mean±SD	Min-max	Reference range*
RBC (10^12^/l)	6.4± 0.69	4–8.2	5.5–8.5
HCT (%)	43.6 ± 4.56	30.3–53.9	37–55
Hb (g/dl)	15.3 ± 1.78	9–18	12–18
MHC (pg)	24.2 ± 2.52	17.6–30.8	19.5–24.5
MCHC (g/dl)	35.2 ± 3.24	28.3–46.4	32–36
MCV (fl)	68.8 ± 4.32	60.9–78.4	60–77
PLT (10^9^/l)	290.2 ± 156.23	44–757	200–500
WBC (10^9^/l)	17.5 ±7.60	8.5–44.9	6–17
BA%	0.9 ± 0.78	0–3	0–2
EO%	9.4 ±7.52	0.3–32.3	0–8
LY%	19.8 ± 9.01	6–41.6	12–30
MO%	4.4 ± 2.81	0.5–10	2–4
NE%	64.5 ± 11.03	40.9–85.8	62–87
BA (10^9^/l)	0.2 ±0.17	0–0.5	0.0–0.4
EO (10^9^/l)	1.7 ± 1.62	0.1–6.5	0.0–0.8
LY (10^9^/l)	3.3 ± 1.74	0.9–9.2	1.0–4.8
MO (10^9^/l)	0.7 ± 0.48	0.1–1.9	0.15–1.35
NE (10^9^/l)	11.6 ± 6.39	5.6–38.5	3.0–12.0

*Internal laboratory reference values; dogs *n*=32

**Table 3:** Mean value for biochemical results in *Acanthocheilonema reconditum* microfilaremic dogs

Parameter	Mean ±SD	Min-max	Reference range*
BUN (mmol/l)	3.93 ±1.68	1.7–8.2	2.5–7.2
Creatinine (umol/l)	74.43 ± 23.36	18–130	40–130
ALT (U/l)	29.9 ± 8.33	10–47	10–45
ALP (U/l)	59.9 ± 33.52	21–173	20–150
Serum total proteins (g/l)	75.83 ± 8.14	58–94	57–77
Albumins (g/l)	34.12 ± 5.78	17–46	25–40
Globulins (g/l)	42 ± 8.59	28–67	25–45
A/G ratio	0.86 ± 0.26	0.30–1.4	0.5–1.3

*Internal laboratory reference values; dogs *n*=43

Statistically, among the examined factors, only the dogs’ living province (*p*<0.0001), the type of hunted game (*p*=0.0002), and ectoparasite infestation history (*p*=0.018) affected the probability of being *A. reconditum* infected (Table 4).

**Table 4:** Risk factors associated with *Acanthocheilonema reconditum* infection in hunting dogs from southern Italy (*n*=2930)

Risk Factor	χ^2^	Df	*p*	OR	95% CI
Living province (ref: Salerno)	35.0	5	<0.0001		
Napoli				5.4	2.1–14.0
Caserta				5.1	2.5–10.6
Avellino				1.4	0.6–3.3
Benevento				1.1	0.4–2.9
Potenza				–	–
Type of game (ref: Birds)	14.3	1	0.0002		
Mammals				2.8	1.6–4.8
Ectoparasite infestation (ref: No)	5.6	1	0.018		
Yes				1.9	1.1–3.1

*χ*^*2*^ Chi square; *Df* degree of freedom; *p* p value; *OR* odds ratio; *CI* confidence interval

**Fig. 4:**
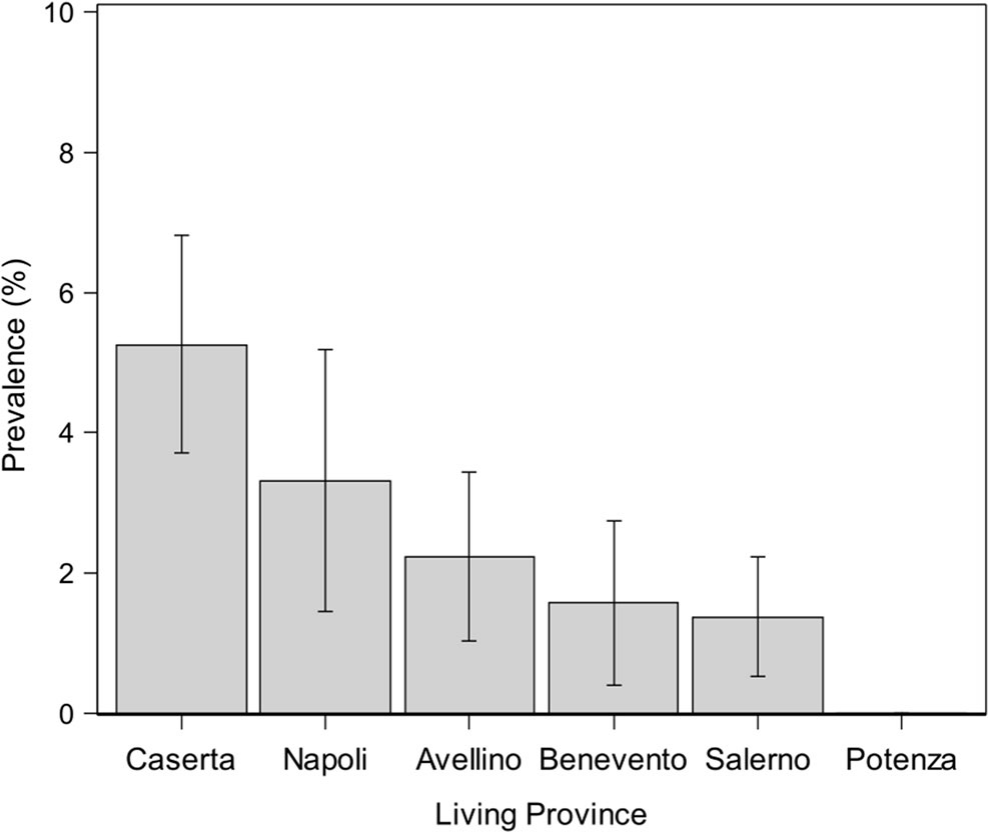
Prevalence (±SD) of *Acanthocheilonema reconditum* infection in hunting dogs from different provinces of Southern Italy

**Fig. 5:**
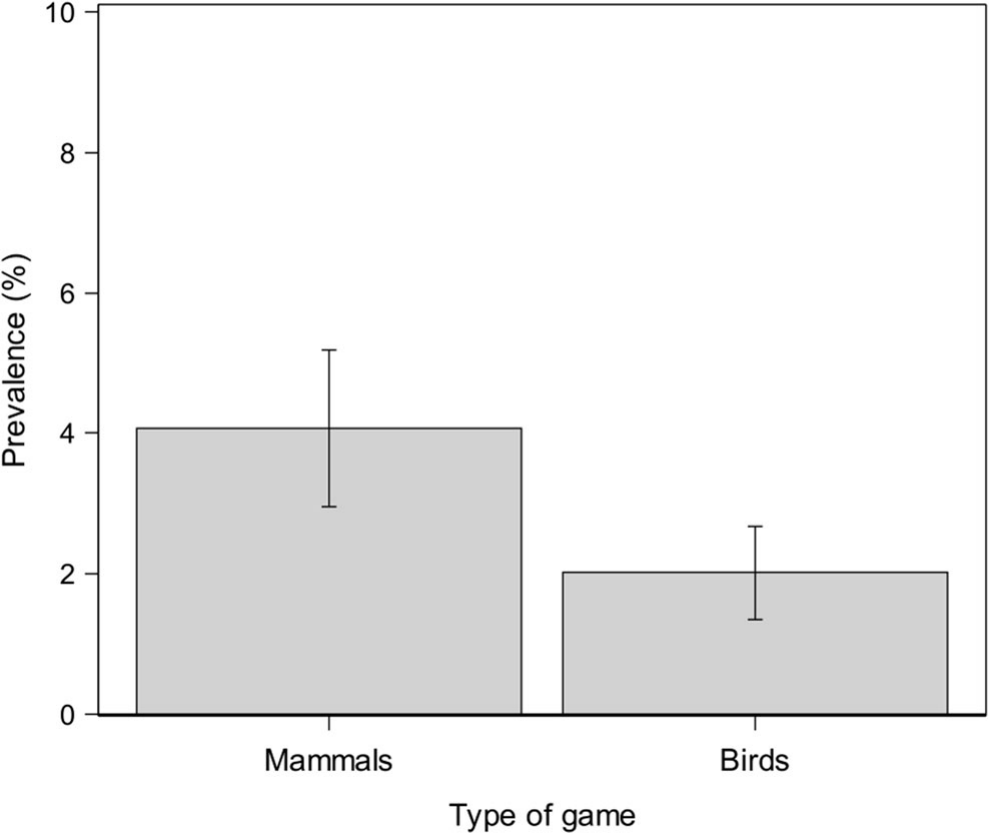
Prevalence (±SD) of *Acanthocheilonema reconditum* in hunting dogs by type of game hunted

**Fig. 6:**
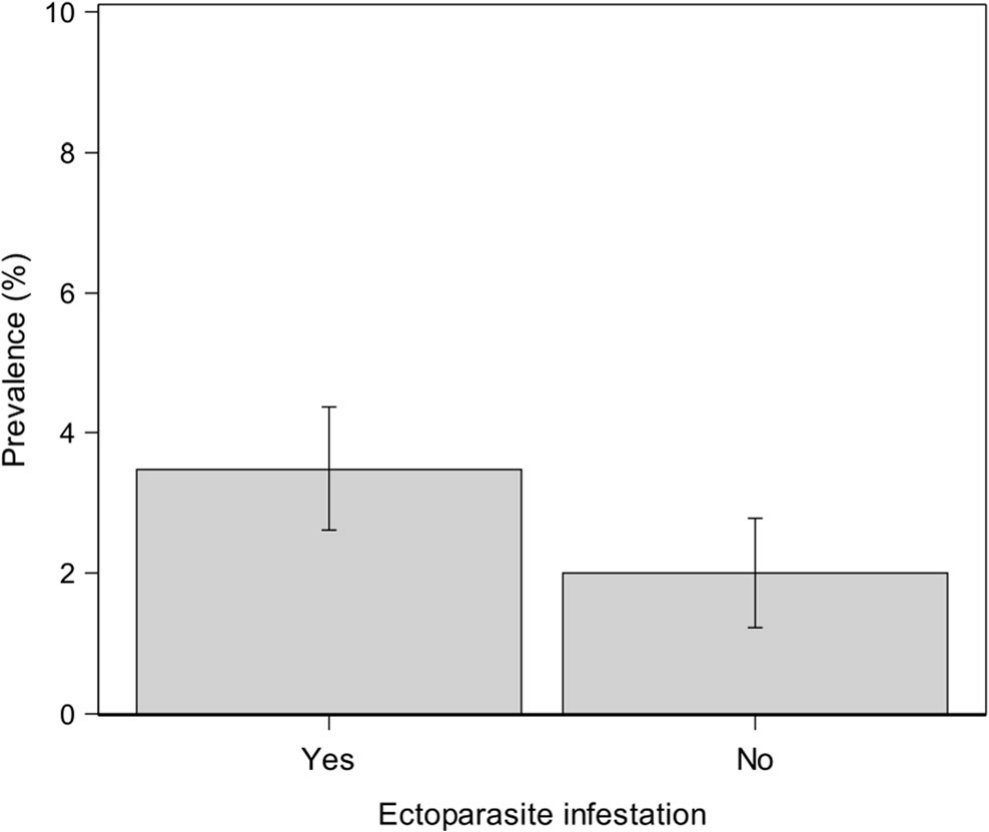
Prevalence (±SD) of *Acanthocheilonema reconditum* in hunting dogs with or without an ectoparasite infestation history

Specifically, living in Napoli (OR=5.4, 95% CI: 2.1–14.0) and Caserta province (OR=5.1, 95% CI: 2.5–10.6), wild mammals hunting (wild boars, hares, and foxes) (OR=2.8, 95% CI: 1.6–4.8) and ectoparasite infestation history (ticks, fleas, and lice) (OR=1.9, 95% CI: 1.1–3.1) represent risk factors for *A. reconditum* infection (Table 4; Figs. 4, 5, and 6).

**Fig. 7:**
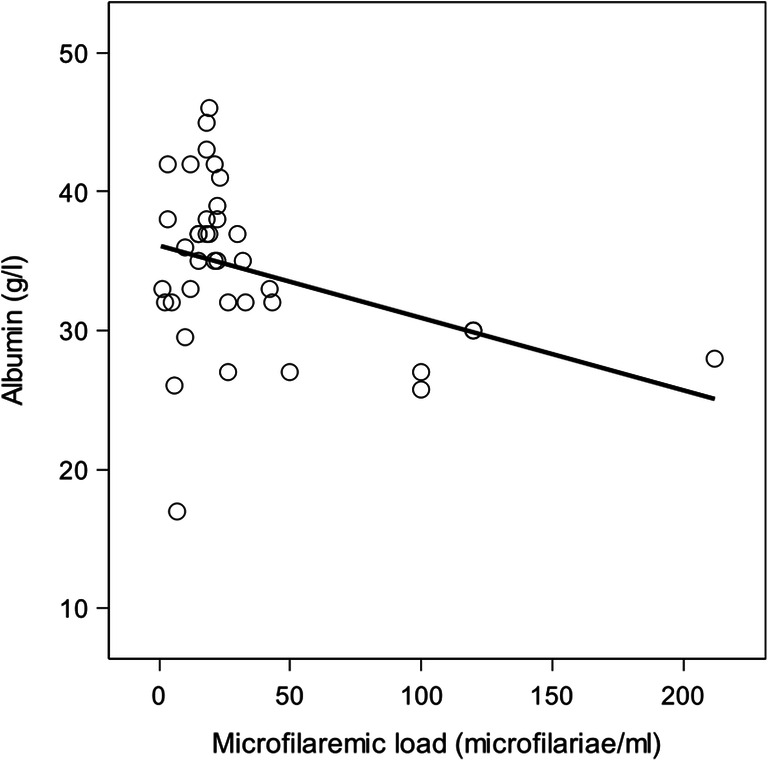
Relationship between microfilaremic load and albumin serum concentration in hunting dogs showing *Acanthocheilonema reconditum* single infection

Concerning the clinicopathological parameters, there was a significant negative correlation between microfilaremic load and the serum albumin levels (Pearson’s correlation coefficient: −0.37; *p*=0.021) (Fig. 7). As the microfilariae number increased, the albumin level decreased linearly. For example, a dog with 100 microfilariae will have significantly lower albumin level than a dog with only microfilariae counts of two, despite the albumin value being within the laboratory reference range. There was no correlation with any other haematological or biochemical variable.

## Discussion

Our study confirmed the presence of *A. reconditum* in hunting dogs living in Campania region of southern Italy. Of the 3020 hunting dogs tested for *A. reconditum*, 84 were microfilaremic with an overall prevalence of 2.8%. In a previous survey, Cringoli et al. (2001) reported a higher prevalence (16.5%; 58/351) in a no specific dog population of the same region. This discrepancy could be explained by the smaller size of the analyzed sample, the concentration of the enrolled dogs in a limited area of the Campania region (Napoli province), and the greater knowledge of the owners about the prophylaxis against ectoparasites developed in the last 20 years. Similar to our results, in a more recent survey in Molise, a small central-southern region close to our study area, Gizzarelli et al. (2019) reported an *A. reconditum* prevalence of 2.5% (8/318) in hunting dogs. Finally, a country-wide dog Filaridae study in Italy demonstrated a lower *A. reconditum* average prevalence of 0.8% (14/1748 dogs tested) (Brianti 2018). From the questionnaire analysis in our study, approximately 30% of hunters reported sporadic extra-label use of ivermectin. Since this macrocyclic lactone suppresses *A. reconditum* microfilaraemia (Lindemann and McCall 1983), it is possible that this analyzed population contained Knott’s test undiagnosed cases.

It is also important to underline that to date, the distribution of *A. reconditum* could be underestimated because it is generally identified during the search for other filarial species. Moreover, the routine use of in-clinic rapid tests for the detection of *D. immitis* antigens in blood samples has facilitated the diagnosis of this major filarial worm in dogs, but has likely contributed to a reduction in the opportunity to diagnose *A. reconditum* infections through the Knott’s test that is a time-consuming procedure (Magnis et al. 2013; Evans et al. 2019). Recently, the molecular methods that allow to achieve a diagnosis at species level for *A. reconditum* have increased compared to testing modalities used in the past (Laidoudi et al. 2020a, 2020b), but as they are cannot yet be considered a routine diagnostic resource.

The history of ectoparasite infestation and hunting of wild mammals are risk factors for *A. reconditum* in hunting dogs. These findings could be explained by the close contact that occurs between wild mammals and dogs during hunting activities, which exposes the dog to the ectoparasites infesting the prey. A recent study described *C. felis* and *C. canis* as most common infestations of wildlife, highlighting that sharing habitats between mammals of different species facilitates the ectoparasite spillover (Clark et al. 2018). The spread of filariasis within wild canid populations, such as foxes, has been well documented in Europe (Hodžić et al. 2015; Ionică et al. 2017). In central Italy, the presence of *A. reconditum* in fox populations reported a prevalence of 10.9% (Marconcini et al. 1996) and 9.1% (Magi et al. 2008), resulting the most common filariasis identified among investigated foxes. Furthermore, the spread of *A. reconditum* is related to its presence in confined populations, thus probably indicating that the infected animals may play a key role as reservoir (Brianti et al. 2012). In fact, the latter authors during a 2-year study reported an *A. reconditum* prevalence of 11.2% in a confined shelter dog population, with an annual incidence of 5.9%. This illustrates how close contact with *A. reconditum*-infected hosts is crucial for the development of new cases, because only the adult flea stage is a competent vector for transmission. Therefore, the proximity, during hunting activities of dogs with wild animals, which are potential reservoirs of *A. reconditum*, could play an important epidemiological role in transmission (Diakou et al. 2016). It should also be added that dogs used for hunting mammals (mainly wild boars) are maintained in packs of at least 4–5 animals (Sgroi et al. 2020), a factor that can favour the spread of the ectoparasites among dogs reared together and thus could facilitate the transmission of *A. reconditum.*

There were significant differences between geographical areas, with higher prevalence in dogs from Caserta and Napoli provinces of Campania region. These results may be related to different dog management practices and to environmental factors as well. For instance, in Caserta province more than one microfilaremic dog belonged to the same owner and most microfilaremic dogs did not receive adequate ectoparasiticide treatment program during the year. In fact, the owners limited the administration of generic spot-on solutions only during the warmer period, leaving dogs exposed to ectoparasites especially during the hunting season. In Napoli province, the high presence of *A. reconditum* had been previously reported, suggesting a localized hotspot (Cringoli et al. 2001). Napoli province was indeed the highest risk, since *A. reconditum* prevalence was relatively high despite only a few dogs having an ectoparasite history, none of which hunted mammals, contrary to Caserta where these risk factors were well represented. It is important to underline that in Campania region the presence of suitable vectors is well documented: Rinaldi et al. (2007) described *C. felis* as the most common flea species infesting the dog population, even in urban areas, reporting that the climatic conditions would easily support the presence of fleas all year long.

Regarding the limited clinical, haematological, and serum biochemical findings generated for the 71 *A. reconditum*-infected dogs, this parasite appears to be minimally pathogenic. Specifically, during the clinical examination, we did not observe any skin lesions (e.g. dermatitis, alopecia and nodules), as previously reported by Brianti et al. (2012). Two *A. reconditum* microfilaremic dogs were reported by owners to have poor performance during hunting activity. Exercise intolerance is a clinical phenomenon that is often observed in dogs during the hunting season (Hunt et al. 2018). It can be difficult to establish the cause of episodic weakness, as it can be related to several pathological conditions, including heartworm disease, which is always considered in the differential diagnosis by clinicians. Papazahariadou et al. (1994) reported a significant correlation between episodic weakness and *D. immitis* microfilaraemia in hunting dogs living in Greece, but this association was not confirmed by authors for *A. reconditum* microfilaremic dogs.

Concerning the limited haematological variables among infected dogs, our results identified leukocytosis with eosinophilia and monocytosis. These findings are consistent with data reported during the acute and chronic phase of an experimentally induced infection (Lindemann et al. 1983). Similarly, Hashem and Badawy (2007) reported leukocytosis, with neutrophilia, eosinophilia, lymphocytosis, and monocytosis in dogs naturally infected with *A. reconditum*. Leukocytosis, primarily involving eosinophils, monocytes, and neutrophils, can be related to the dog’s immune response to cuticular microfilarial antigens (Simon and Genchi 2001).

A finding of increased serum total proteins and total globulin levels in our study population is consistent with findings reported by Hashem and Badawy (2007), and can be explained by a chronic immune response to the parasite or perhaps to other undiagnosed infections. *Acanthocheilonema reconditum* microfilaremic dogs in our study had no consistent indications of liver or kidney injury, as instead previously reported by Hashem and Badawy (2007).

Hypoalbuminemia has been reported in *D. immitis* and *D. repens* infections because of liver and kidney damage due to immune-pathological and mechanical activity of microfilariae (Mircean et al. 2017). Liver injury was a speculated cause for hypoalbuminemia (Hashem and Badawy 2007); however, when hypoalbuminemia occurs in natural *A. reconditum* infections, it is likely due to another cause. Hypoalbuminemia was not detected by Lindemann et al. (1983) in experimental *A. reconditum* study, despite documented proteinuria during the chronic infection phase. In the current study, there was a significant negative correlation between microfilaremic load and serum albumin level. This finding could be explained by the nutritional utilization by the microfilariae of amino acids from the bloodstream (Simon and Genchi 2001; Simón et al. 2012) or alternatively other mechanisms. Among microfilaremic dogs in this study, only a single animal was hypoalbuminemic, whereas the albumin concentrations of the other dogs, although negatively related to the parasite load, remained in the reference range, suggesting a body response to the presence of microfilariae but not a real pathological effect.

## Conclusions

In conclusion, the present study further confirms the circulation of *A. reconditum* infection in southern Italy and highlights how hunting dogs represent a risk population due to their outdoor lifestyle and their close contact with wildlife. Moreover, regarding this filarial infection, further studies should be performed to better investigate epidemiological relationships between hunting dogs, wild animal populations, and the biological role of the different competent vectors sharing the same area. Finally, our data highlight that *A. reconditum* can be considered a minimally or non-pathogenic filarial infection in dogs. However, as some dogs may have a high microfilarial load, it is important to adhere to preventive measures against arthropod vectors to limit the infection with this filarial worm.

## Data Availability

The data supporting the conclusions of this article are included within the article.
